# The unconscious and the transcendental: Husserlian phenomenology in intersubjective systems theory

**DOI:** 10.3389/fpsyg.2023.1282640

**Published:** 2023-11-29

**Authors:** Rozemund Uljée

**Affiliations:** Institute for Philosophy, Humanities, Leiden University, Leiden, Netherlands

**Keywords:** psychoanalysis, phenomenology, relational turn, Husserl, transcendental, intersubjectivity

## Abstract

The relational turn in psychoanalysis can be identified by the replacement of Freudian drives for intersubjectivity as main regulative principle. “Intersubjective systems theory” is the name of one strand within the relational turn that explicitly locates its philosophical foundations in a number of phenomenological insights. In this paper, I investigate some of the essential phenomenological assumptions underlying intersubjective systems theory. I identify 2 main problems: 1. intersubjective systems theory relies on the premise that meaning is intersubjectively constituted, yet fails to offer an account of this constitution; 2. intersubjective systems theory is based on an ambiguous conception of the unconscious. The aim of this paper is to show how Husserlian phenomenology offers a valuable theoretical foundation for intersubjective systems theory in the sense that it presents a convincing account of the intersubjective constitution of meaning that in its very constitution allows for a dynamic and situational relation between consciousness and unconsciousness.

## 1 Introduction

Theorists working within the relational field have a broad and diverse understanding of what is meant by the relational turn. They do, however, share one main theoretical assumption: all put an “emphasis on the centrality of relatedness” (Mills, 2005, 157). In itself, the theme of relatedness is not new to psychoanalysis: Freud, in many places in his work, makes the point that the self is driven by needs, desires, and developmental processes in an intersubjective context (Reisner, 1992; Frank, 1998). Later, object relations theorists like Klein (and others) emphasised even more the point that every aspect of life is governed by relatedness. Nonetheless, what is distinctive about the relational turn is the actual *replacement* of Freudian drives for intersubjectivity as general regulative principle (Mitchell, 1988; Stolorow and Atwood, 1992). Whereas in Freud, unconscious drives are viewed as the main motivational factor for behaviour, within the relational turn it is intersubjectively constituted meaning that regulates experience.

In very general terms, theorists who view their work as belonging to the relational turn assume that intersubjectivity is privileged over the distinction between subjectivity and objectivity, which, however does not imply that the existence of individual subjects, objects and a world is renounced. Even though the term “intersubjectivity” is widely used in many different psychoanalytical contexts, it still remains relatively unclear what exactly is meant by it. In philosophy, the question *how* the relation between intersubjectivity, subjectivity and world plays out, is still widely debated, let alone in psychoanalysis. This is why Jon Mills accuses the field of contemporary psychoanalysis of remaining “naïve to formal metaphysics” (Mills, 2005, 159).

Nevertheless, with regards to the question of intersubjectivity, two main tenets within the psychoanalytic literature can be identified: the *developmental* view and the *systems* view (Mills, 2005, 159). Both approaches can be present simultaneously at different levels. Stern (1985), Benjamin (1988), Mitchell (2000), and Beebe and Lachmann (2003), among others, conceive of intersubjectivity in developmental terms: as the learning process that is about acknowledging the existence of the internalized other, be it the mother, father or the analyst. Where the developmental view echoes Hegel’s master slave dialectic in the sense that intersubjective recognition is viewed in terms of a success and a result of a developmental process, the systems view finds its philosophical inspiration in phenomenology.

Obviously, phenomenology and psychoanalysis are distinct disciplines with their own conceptual framework and guiding questions. But considering the historical trajectory of both psychoanalysis and phenomenology, it is no surprise that the developments of respective theories intertwined. Both Freud and Husserl attended Brentano’s lectures in Vienna (Freud, 1909/1990); theorists such as Binswanger and Jaspers sought to combine the insights from the different disciplines in their phenomenological approach to psychopathology. At present, there is a general agreement that phenomenology can offer psychoanalysis a fitting paradigm for its analysis of subjectivity and its disturbances (Fuchs et al., 2019). A number of reasons can be offered for this: 1. phenomenology’s focus on and terminology regarding pre-reflexive phenomena are adequate to offer a more profound understanding of emotional disturbances (like existential feelings, atmospheres); 2. Phenomenological conceptions such as embodiment and attunement offer an interpretation of the therapeutic relation in terms of an embodied encounter. 3. Phenomenology does not seek to describe subjectivity in terms of objectivity, but in terms of ways in which the world discloses itself to it. In this sense, its focus is on the way experience is structured rather than as an object to be described.

Intersubjective systems theorists like Stolorow and Atwood are explicit about their commitment to phenomenology, and compared to the majority of other relational theorists, they share not only many moving words of wisdom about their clinical experiences, but offer by far the most extensive and detailed philosophical framework (Mills, 2005). They view phenomenology as more than useful terminology; it is their main source of inspiration: both as a theoretical model and as a clinical method. Their phenomenologically inspired conception of relatedness means they conceive of intersubjectivity as an ontological category of interrelated subjectivities that together form constellations of meanings. Stolorow and Atwood are convinced that, viewed from an intersubjective paradigm, all of the “clinical phenomena” that are matters of interest for psychoanalysis, are perceived to take place within “systems of interacting, differently organized, mutually influencing subjective worlds” (Stolorow and Atwood, 2019, 66).

In this paper, I investigate some of the essential phenomenological assumptions underlying the intersubjective view that Stolorow and Atwood present. A legitimate question one might ask is: why does this matter? In answer to this point, it must be remarked that to the extent that psychoanalysis does not only consist of a body of theoretical literature, but also a clinical practice, the results within clinical practice cannot be viewed in isolation of its theory. The *form* of the relational practice has been applauded by many (Bacal, 1998; Greenberg, 2001; Mills, 2005) for the human, sensitive manner of treatment that has room for a personal experience rather than a standardized approach to therapy. Obviously, these are noted strengths. The actual *content* of the clinical relational approach is about making new meanings together. Whereas Freudian psychoanalysis is concerned with insight and interpretation, the, the aim of relational psychoanalysis is the co-creation (between clinician and patient) of meaning constellations, not in order to erase the old and harm- or pain-ful ones, but to find a new (and less overwhelming) relation to them.

Intersubjective systems theory repeatedly states *that* meaning is intersubjectively constituted. However, *how* this process takes place, and consequently, how intersubjectively constituted meaning constellations can be altered in a clinical setting, remain unclear. My focus will be on what I call a transcendental poverty with regards to the intersubjective constitution of meaning that intersubjective system theory relies on. In this paper, I argue that this transcendental poverty is the outcome of two fundamental problems regarding Stolorow’s and Atwood’s phenomenological commitments. The first problem concerns the supposedly intersubjective constitution of meaning. Although phenomenological thinkers like Husserl, Merleau-Ponty, and Levinas have written explicitly and extensively about the constitution and significance of intersubjectivity, Stolorow and Atwood are convinced that Heidegger’s *Being and Time* offers them the most adequate phenomenological framework from which to think the embeddedness of (emotional) meaning. This is already in itself remarkable, since Heidegger has been heavily criticized from many different sides for what is perceived a lack of regard for the other person (and a corresponding ethics) throughout his works. But especially with regard to intersubjective systems theory, Heidegger’s *Existential Analytic* is problematic in the sense that it does not offer an account of the intersubjective constitution of meaning, which is the main premise of intersubjective systems theory.

The second problem concerns confusion about the role of the unconscious in the constitution of meaning. This confusion can be traced back to the fact that intersubjective systems theory maintains an ambiguous relation to Freudian psychoanalysis. On the one hand, a number of fundamental Freudian insights, such as transference and repression, take center stage in the clinical setting and the theoretical framework. On the other hand, the adherence to phenomenology as a science of consciousness opens the question to what extent it is still possible to place intersubjective systems theory in the psychoanalytical tradition.

The main aim of this paper consists in showing that, in light of the problems mentioned above, the thought of Husserl offers a convincing theoretical framework for intersubjective systems theory. Within this context, intersubjectivity is not conceived as an empirical reality, but as a transcendental core concept, offering an interpretation of the term in which subject and the other are co-originated. Furthermore, I clarify how any constitution of meaning always already relates to what is not conscious. As such, I argue that that Stolorow’s and Atwood’s philosophical commitments are much closer to the thought of Husserl than they assume, and that Husserl’s account of transcendence and the transcendental offers a convincing philosophical ground for intersubjective systems theory.

I begin by outlining Stolorow’s and Atwood’s relation to phenomenology, specifically their relation to Heidegger. I point out what I think are some fundamental problems that result in the transcendental poverty noted above. I then turn to Husserl’s and his account of the transcendental. I argue that Husserl initiates a thought of co-determination between subject and other in which neither is ordinary. Thirdly, I discuss the place and role of the unconscious in Freud, intersubjective systems theory, and Husserl, in order to show that Husserl’s dimension of the unconscious, as a “thing” of consciousness, offers intersubjective systems theory the possibility to conceive as the unconscious as a dynamic and situational limit. I conclude with some remarks about the temporality that Husserl introduces and what its possible implications might be for clinical practice.

### 1.1 Intersubjective systems theory and phenomenology

Stolorow and Atwood view the Freudian conception of the mind as committed to a Cartesian mind–body dualism, which conceives of the mind and the world as two independent terms and would thereby imply a “decontextualization” of mind and world (Stolorow, 2011, 13). They regard such a conception as highly problematic: even though Freud’s view of the mind includes the unconscious, it still views the subject as “passive receptor of discrete atomic impressions from the outer world.” Stolorow and Atwood criticize the interpretation of consciousness as “a quasi-spatial container.” Such a conception of mind and world necessarily involves “a projection *into* experience of the qualities of material objects *of* experience,” and reflects “a failure to confront the attributes of subjectivity in their own distinctive terms” (Stolorow and Atwood, 2014, 12). Stolorow and Atwood call for the urgent move away from this decontextualization, which they view as the gesture of metaphysicalizing of subjectivity, and interpret their work as an attempt to liberate “psychoanalytic theory and practice from various terms of Cartesian, isolated mind-thinking en route to a post-Cartesian psychoanalytic perspective” (Stolorow and Atwood, 2014, 113). Such a liberation can take place through the development of intersubjective systems theory as a phenomenological contextualism.

As noted, phenomenology can operate in two ways: both as a theoretical foundation and as a clinical method. The theory is phenomenological in the sense that it seeks to illuminate “organizations or worlds of emotional experience; contextual in the sense that “it holds that such organizations of emotional experience take form, both developmentally, and in the psychoanalytic situation, in constitutive relational or intersubjective contexts” (Stolorow and Atwood, 2014, 113). With regard to theory, Stolorow and Atwood’s phenomenological approach offers a departure from metaphysical conceptions of consciousness, and towards relational contexts, and thus from what they call the “intrapsychic to the intersubjective” (Stolorow and Atwood, 2019, 66). With regard to method, they approach their own clinical practice as a form of phenomenological enquiry in which the task at hand is to illuminate the world as it is presented to the patient by the meaning structures of the patient.

Intersubjective systems theory operates like philosophical phenomenology in the sense that the point of departure of both theories is the development of an independent science, of experience, with its own terms and methodology. Yet, they can (obviously) not be conflated. Philosophical phenomenology concerns itself with the constitution of universal meaning structures in and for subjectivity. It is only through careful reflection by the phenomenologist that these meaning structures can be elucidated. In doing so, the phenomenologist has, according to Stolorow and Atwood, “essentially defocused the individualization of a world in the quest for knowledge of subjectivity in universal terms” (Stolorow and Atwood, 2014, 8). Intersubjective systems theory however, investigates the unique world of experience of a patient and is based on the dialogue; a setting that a philosophical phenomenologist would reject as operating from within the natural attitude (Togni, 2018, 77).

Reflecting on their theoretical and clinical development throughout the years, Stolorow and Atwood find that it mirrors the philosophical trajectory from classical phenomenology to what they call contextualism: they write that a “dedication to phenomenological inquiry” led them to recognize the context-embeddedness of all emotional experience. They equate their journey with the movement from Husserl’s Cartesian phenomenology to what they call “Heidegger’s phenomenological contextualism” (Stolorow, 2011, 19). Husserl is supposed to represent the isolated mind-thinking that they want to depart from, and Heidegger stands for the radical context-embeddedness of all experience that is assumed to underlie their claims.

Given the comparison between Husserl and Heidegger, it does not come as a surprise that Stolorow and Atwood describe Husserlian phenomenology in harsh terms. Their criticism focuses on the standpoint from which transcendental phenomenology is conducted: the reduction. They perceive a methodological lack of clarity in the performance of the reduction: here, the phenomenologist conceives of herself as the pure subject of her intentionality, and thus as a theoretical spectator of the transcendental conditions of the possibility of experience. They ask, however, whether the act of this reflection not itself an experience? Stolorow and Atwood criticize transcendental phenomenology for assuming that it is possible to step out of the factical situation of subjectivity, into a “realm of presuppositionless certainty” about the essence of consciousness (Stolorow and Atwood, 2014, 12). Such a gesture would deny human finitude as well as the fact that knowledge is a “social creation” (Stolorow and Atwood, 2014, 12). Moreover, the reduction would be in contradiction with itself, since it cannot include all unconscious and thereby unsuspended presuppositions. Husserl, they find, conceives of the transcendental subject as an isolated product of an isolated method, for whom the world is nothing more than a “correlate of its inner intentions” (Stolorow and Atwood, 2014, 12). Consequently, transcendental phenomenology is a “spectacle of thought” that is “detached from social life” and circles “inwardly upon itself,” and thereby mistaking “a reified symbol for its own solitude for the discovery of its absolute foundation” (Stolorow and Atwood, 2014, 12). Stolorow and Atwood note that in this manner, Husserl’s movement of the reduction eliminates the unique essence of an individual’s experience of the world, which is precisely what is most important in the psychoanalytic situation (Stolorow and Atwood, 2014, 11).

### 1.2 Heidegger, temporality, and transcendental poverty

Stolorow and Atwood are a lot more appreciative of Heidegger’s approach to phenomenology and although Heidegger’s question of the meaning of Being in general is outside the domain of relevance for psychoanalysis, they view his Existential Analytic as the phenomenological foundation for intersubjective systems theory. This is so because Heidegger, according to Stolorow and Atwood, manages to close, “the ontological gap between human Being and its world” (Stolorow, 2011, 6). Like phenomenological contextualism, Heidegger’s thought is concerned with conceptions of human beings as *experiencing* beings. Moreover, Heidegger understands that a human being’s interpretation of itself in terms of *quidditas*, prevents its own self-understanding *as* a human being.

With regard to Heidegger’s Being-in-the-world, Stolorow and Atwood are correct by stating that Being-in-the-world can be conceived as a radical contextualism that “unveils the basic structure of our human kind of Being as a rich contextual whole” and that reveals that the human being is “saturated with the world” in which it dwells; that the world we inhabit is “drenched in human meanings and purposes” (Stolorow, 2011, 18). In other words, Heidegger’s fundamental insight is of central importance to intersubjective systems theory: the human being cannot be satisfactorily understood in isolation from its world. Consequently, Heidegger’s thought is supposed, on the one hand, to ground the existential significance of trauma (or: the recognition that trauma is embedded into the basic constitution of human existence), and, on the other hand, shows that emotional trauma is ontologically revelatory in the sense that trauma can disclose authentic existence. Although these are legitimate strengths of Heidegger’s thought, and in line with Stolorow’s and Atwood’s contextualism, the transcendental poverty that intersubjective poverty suffers from can at least in part be attributed to Stolorow’s and Atwood’s reliance on Heidegger’s existential analytic. This, I think, is due to three reasons, that I list here.

The first reason is that Stolorow and Atwood seem to have a confused conception of temporality. They follow Heidegger’s account of radical finitude. Yet, they present one fundamental modification of Heidegger’s existential analytic as presented in *Being and Time*: they propose that Being-towards-death always already includes Being-toward-loss, which amounts to stating that death and loss are “existentially equiprimordial” (Agosta, 2010). The gesture of relationalizing finitude offers an account of shared solidarity and “kinship-in-finitude” between subjects. Such a relation is supposed to entail “a reciprocal co-disclosure of our common finitude” and is referred to in terms of “emotional kinship in the same darkness” (Stolorow, 2011, 77–78). Stolorow and Atwood’s gesture of adding loss and grief to the domain of the ontological is something that Heidegger would reject, since for Heidegger the loss of the other belongs to the ontic, and it is different from the ontological loss of Being. Heidegger states this explicitly in *Being and Time*: “In suffering this loss [of the Other], however, we have no way of access to the loss-of-Being as such which the dying man ‘suffers’, The dying of others is not something we experience in a genuine sense” (Heidegger, 1962, 282).

With this gesture, Stolorow, and Atwood thus already step outside of Heidegger’s Existential Analytic in the sense that with the question of loss, and with the possibility of surviving the other’s death, they are no longer subscribing to finitude as the horizon for meaning, which is precisely Heidegger’s central claim. Loss, and the possibility of surviving a beloved other, as shown convincingly by thinkers like Derrida and Levinas already introduces, beyond the finitude of Being, a temporality of infinity: of a domain of meaningfulness that can be present beyond my death, for those who might come after me. The intertwining of finitude and infinity in the constitution of meaning is not addressed by Heidegger, but by Husserl, as I seek to describe below.

The second cause of the transcendental poverty is that Heidegger cannot offer Stolorow and Atwood a theory of constitution. This is so because the point of departure for Heidegger’s philosophical project is the world as always already infused with meaning, something of which we become aware essentially in anxiety, since, “As one of Dasein’s possibilities of Being, anxiety, together with Dasein itself as disclosed in it provides the phenomenal basis for explicitly grasping Dasein’s primordial totality of Being” (Heidegger, 1962, 227). One can wonder whether that means that Heidegger’s phenomenological approach did not adequately appreciate the reduction, so that he remained operating from within the natural standpoint. Yet, insofar as the reduction entails a gesture of “putting reality out of play,” precisely because the phenomenological purpose is to disclose the phenomenal “being-sense” of reality itself (Seeburger, 1975, 215), Husserl and Heidegger are remarkably close (even though Heidegger barely addresses the reduction in *Being and Time*). Their fundamental disagreement then is not so much about the question whether the reduction is possible or desirable, and thus whether reality can be suspended in this manner, but about what is at stake in such a suspension. For Heidegger, the reduction entails the gesture of revealing the relationship between man and world from *within* that very relationship. Or, in Heidegger’s words, the world as totality of present-at-hand must be suspended so that the worldliness of the world can announce itself: become a phenomenon available for description (Seeburger, 1975, 215). The main point here is that, according to Heidegger (and Stolorow and Atwood appreciate Heidegger precisely for this fact), phenomenology cannot be without presuppositions, and is therefore a kind of hermeneutics. In other words: philosophy always already presupposes the human beings’ engagement with the world. Phenomenology can never escape the hermeneutical circle (Seeburger, 1975, 215; Heidegger, 1962, 7). Thus, whereas for Husserl, as I will seek to point out, transcendental consciousness must be conceived as the source of the constitution of meaning, for Heidegger, the human being’s existential structures disclose meaning as always already there. As such, Heidegger’s thought does not offer an account of the constitution of meaning, which is precisely the main theoretical premise of intersubjective systems theory.

The final point here is related to the question of constitution and concerns the question of intersubjectivity. With regard to intersubjective systems theory, the problem with its reliance on Heidegger is not so much the fact that meaning-making takes place within an ontological context. But rather the fact that ultimately, meaning is disclosed from within the solitude of one’s unsubstitutable Being-towards-death. It is true that for Heidegger, Being-in-the-world is always already Being-with, but the relationship between Dasein and the other is mediated by Being. Or, in other words: Dasein and the other are engaged with one another by relating to Being together. Dasein is essentially Mitdasein, but it is in its isolation of the others that Dasein can engage with its source of meaning (i.e., Being). As such, Mitdasein, in Husserl’s terms, does not have a constitutive character. One might respond by stating that Stolorow and Atwood attempt to enrich Being-towards-death with Being-toward-loss, thereby, in their own terms, “relationalizing Heidegger,” yet it still is the case that Heidegger does not offer an account of the intersubjective constitution of meaning. He has very good reasons for that, since from his perspective, doing so would amount to a metaphysical gesture, where thinking of Being would be more originary. Intersubjective systems theory however needs an explanation of the intersubjective constitution of meaning in order to ground it, and it cannot find it in Heidegger.

Whether Stolorow and Atwood view intersubjectivity as Mitdasein or in empirical terms, they remain, despite their claims, within a fundamental isolationist paradigm when they write that “psychoanalysis is pictured […] as a science of the intersubjective, focused on the interplay between the differently organized subjective worlds of the observer and the observed” (Stolorow and Atwood, 2014, 34) This phrase shows that Stolorow and Atwood assume that intersubjectivity is derived from subjectivity in ways that are self-defeating for their project. In fact, a similar point can be made regarding Freud’s account of relationality: it is true that the Freudian subjects is fundamentally relational in the sense that it has relations with others. However, the issue is that in Freud, meaning is not intersubjectively constituted, but the outcome of personal drives.

### 1.3 The transcendental in Husserlian phenomenology

In what follows, I seek to make clear that the later Husserl’s interpretation of transcendental constitution is intersubjective in the sense that self and other are co-implicated in the constitution of meaning. As such, it satisfies the main requirement for a philosophical foundation of an intersubjective approach to psychoanalysis: an explanation of the way in which intersubjective meaning is constituted.

It is probably not an exaggeration to state that the difference between transcendence and transcendental philosophy offers the entire paradigm for Husserl’s phenomenology (Caputo, 1979, 205). Here, transcendence is conceived, broadly speaking as that which overflows consciousness, the *plenum*, which, “being other than consciousness, never gives itself up to consciousness” (Caputo, 1979, 205). For Husserl, it is the task of transcendental philosophy to elucidate the conditions of transcendence (Husserl, 2002, 259). Or, in other words, transcendence can be clarified by systematically disclosing constituting intentionality (Husserl, 1950, 34, 65; Zahavi, 2015, 232).

Obviously, the term “transcendental” has a rich philosophical history, and there are specific reasons for the fact that when Husserl labelled his own thought “transcendental,” he was referring to Kant. Husserl gives credit to Kant as the inaugurator of the transcendental tradition, but is also very critical of numerous aspects of Kant’s thought. It therefore matters that we do not to conflate Husserlian transcendental phenomenology with Kantian transcendental philosophy (which, I think, is what Stolorow and Atwood do). Husserl finds that Kant does not have a proper concept of the *a priori* and that the Kantian deduction is “a masterpiece of top-down transcendental reasoning.” Zahavi (2015, 234) that operates with “an overly strict distinction between sensibility and understanding” (Husserl, 1956; Zahavi, 2015, 235). But possibly Husserl’s main point of disagreement with Kant is that it is not possible to assert that something is, without it being given in experience. As such, Husserl denies the existence of the Kantian thing in itself (Husserl, 1956, 232) and berates him for endorsing an epistemology inspired by metaphysics. For Husserl, the question of whether there is a world that is beyond consciousness is a realist assumption that only matters from the standpoint of the natural attitude. Instead, we must place transcendental subjectivity at the origin of meaning-constitution, and considered from a transcendental perspective, there is nothing “outside” of consciousness to which it should relate, since:

*An* absolute reality is no more or less valid than a round square*. “Reality” and “world” here are just headings for certain valid unities of sense, namely, unities of the “sense” related to certain connections of the absolute, pure consciousness* (Husserl, 1983, 129).

The transcendental standpoint can be reached through the phenomenological reduction, which re-conducts phenomena to the constitutive activity of consciousness. While revealing the constituted character of transcendence, the reduction at the same time points to constituting subjectivity as the requirement for transcendence. The goal of the suspension of the external world that is required is not so much to exclude the external world, as Stolorow and Atwood seem to think, but to put into brackets a dogmatic attitude towards it. Or, as Zahavi remarks: the reduction does not “involve an exclusive turn toward inwardness.” Instead, by revealing the dimension of pure phenomenality, the reduction allows us to see the world anew, in “its significance and manifestation for consciousness” (Zahavi, 2015, 234). The reduction must thus surely not be conceived as a process of abstraction from the world, or an invitation to dwell within the poetics of first-person experience. Instead it is a liberation from an abstraction that preceded it, namely, the *Weltthesis*. In this way, the reduction reveals that the world, is a constituted constellation of meanings (Zahavi, 2015, 225). Furthermore, the reduction allows the subject to discover the limitations of the natural attitude in the sense that within the natural attitude, subjectivity is not aware of its transcendental structure. As a phenomenologist, I can, Husserl writes in *Crisis*:

*at any time go back into the natural attitude, back to the straightforward pursuit of my theoretical or other life-interests […]. Thus every new transcendental discovery, by going back to the natural attitude, enriches my psychic life and (apperceptively as a matter of course) that of every other* (Husserl, 1970, par. 59, 210).

In so doing, the reduction offers two differing yet complementary accounts of subjectivity at the same time. On the one hand, it presents a neo-Kantian transcendental ego as the formal structure of all experience. And on the other hand, the reduction reveals how consciousness is always already intertwined with its intentional correlate, and thus always already involved with the world. Because this is the case, Cartesian isolation is radically impossible, not only because Descartes’ questioning is already abstract, but also because an isolated subjectivity is a contradiction. As Eugen Fink puts it: transcendental life is always “*already in the midst of world-constitution*” (Fink, 1995, 58). It is wrong to think of knowledge in terms of a relation between two independent terms, as if consciousness and world only by “coincidence happened to each other” (Husserl, 2003, 30). As such, the main focus of Husserl’s account of phenomenology is not an isolated subject. On the contrary: the subject matter of phenomenology is consciousness precisely because it discloses the world. This is why phenomenology must primarily be understood as seeking to describe the different ways in which the transcendence of the world is given (be it through perception, remembrance, imagination) and to investigate the conditions of possibility of this givenness.

Since the reduction discloses the worldliness of the subject as a constitutive experience, it discloses that very subject as an embodied and social subject (Husserl, 1950, 130; Zahavi, 2015, 240). The later Husserl is very clear about the necessity to include human sociality and culture in the transcendental analysis (Husserl, 1956, 282). As Zahavi points out, in lectures presented in London in 1922, Husserl explicitly states that the development of phenomenology “necessarily implied the step from an “egological” […] phenomenology into a transcendental sociological phenomenology having reference to a manifest multiplicity of conscious subjects communicating with one another” (Husserl, 1981, 68; Zahavi, 2015, 235). It leads Zahavi to conclude that Husserl’s later thought can be viewed in terms of an “explicit defense” of an “intersubjective transformation of transcendental philosophy (Zahavi, 2015, 235).

Here, it is not the question whether transcendental phenomenology offers a proper paradigm from out of which to think intersubjectivity as relation between different subjects. Intersubjectivity is not a term that describes describes a directly observable reality, but operates as a transcendental concept. As such, another route must be taken because it is only through the appropriate elucidation of intersubjectivity that transcendental phenomenology can be understood. It is, as Zahavi notes, only through an explication of transcendental intersubjective sociality as the basis “in which all the truth and all true being have their intentional source” (Zahavi, 2015, 235). Thus, phenomenology is transcendental because it seeks to describe how transcendence can be described constitutively; this can only be done through an elucidation of intersubjectivity:

The transcendence of the world consists in its being constituted by means of others, by means of the generatively constituted co-subjectivity. It is through them that it acquires its ontic sense (Seinnsinn) as an infinite world (Husserl, 2006, 393).

What is meant here? Whereas Husserl initially sought to think a relation between subject and other where they are distinct from one another, in his later thought, he develops an intertwining relationship of co-determination, which, fundamentally means that the reduction that leads us to transcendental subjectivity at the same time means a reduction to the transcendental intersubjectivity made accessible within it (Husserl, 1973, 73–5, 403).

There are four elements of this account of transcendental intersubjectivity that matter with regard to intersubjective systems theory. First of all, the fact that the reduction reveals intersubjectivity offers a rehabilitation of this term from the standpoint of intersubjective systems theory since it precisely discloses the subject in terms of its worldliness. It is strange that that Stolorow and Atwood are so dismissive of Husserl’s reduction especially since a reduction is also required in intersubjective systems theory and within a clinical setting: on the one hand, a suspension of the ontological realm allows on the focus of what are considered meaningful mental states in and for the patient, before even a question about the “truth” or “realness” of these experiences is asked. On the other hand, the subjectivity of the subject is always already viewed in terms of relation: intersubjective dynamics are viewed as constitutive for meaning.

The second is that Husserl offers an account of intersubjectivity as a transcendental concept. It allows him to move beyond binary oppositions, since he convincingly shows that intersubjectivity and subjectivity are co-originary, where on the one hand, only subjects can interact reciprocally, and on the other hand, a subject can only be what it is from a background of intersubjectivity. The one cannot be assumed without the other, nor can the other be assumed without the one. As such, Husserl decisively transforms the hierarchy that he (in his earlier thought) initially subscribed to: that it is not subjectivity at the basis of intersubjectivity. The opposition is transcended in the formulation of the relation between subjectivity and intersubjectivity in dialectical terms: as co-determining. As such, self and other coincide and do not coincide: they are a *Verbundenheit* (Husserl, 1968). Transcendental intersubjectivity is located in the intertwining of self and other; the fact that they are woven together, and thereby structure the transcendence of the world. With regard to intersubjective systems theory, it is thus so that a transcendental account explains the constitution of meaning as intersubjective and removes the confusion regarding the primacy of either subjectivity and intersubjectivity.

The third element is the fact that reality cannot be experienced independent from intersubjectivity. Or, in other words, a subject can only experience the world insofar it is the member of a community (Husserl, 1950, 166). For Husserl, subjective experience is guided by anticipations of what we consider *normal*. As such, normality is thought as a constitutive concept that takes place simultaneously at a subjective and intersubjective level. Therefore, it allows for a coherent and familiar experience, while it also establishes a shared world as the ground for communication. A subject is capable of constituting, experiencing and structuring reality according to the meanings, convictions, and patterns that have already been sedimented in consciousness (Husserl, 1966, 186). As such, consonance is a necessary condition for experience, and already takes place at the level of passive synthesis. In the case that something is not consonant with the previous experience of a subject, but deviant, and thereby a modification of normality, a new normality must be constituted, by either ignoring the deviation or including it into one’s pattern of experiences, and thereby enriching it.

Normality is intersubjectively established through shared practices and experiences, since the different anticipations of consciousness are brought about by a shared world and thus by intersubjectively shared apperceptions (Wehrle, 2018, 56). Deviance of instersubjectively established normality occurs when the experience of a subject is not commensurable with social anticipations: a culture clash in the case of moving to another country is a good example (Wehrle, 2018, 56). One must adapt to the new circumstances in order to enter the shared domain of normality (again). Awareness of such deviations is important, and even a requirement for intersubjective concordance (and thus normality).

Normality can only be established because it is dynamic and because it is fragile: it already points to its opposite in terms of a deviation or abnormality and we can only become aware of what is normal by being confronted with what we think is not. This is what Merleau-Ponty demonstrated in the *Phenomenology of Perception*: we need psychopathology in order to be able to define normality. At the same time, abnormality, as deviation, already points to normality as its ground, which is necessary for a future sense of normality (Wehrle, 2018, 56). Here, normality is viewed as dynamic and context-based. It offers an integrated and intersubjective ground from which to illuminate emotional trauma, and thereby proposes an alternative to reified diagnostics in psychopathology. And is not that precisely what Stolorow and Atwood are after when they write that “there are no psychiatric entities […], only traumatic contexts” (Stolorow and Atwood, 2019, 77)?

The final point is that transcendental phenomenology necessarily deals with historicity: normal life is a generative life: transcendental structures develop over the course of time and can be transformed. One could even say that insofar the transcendental *a priori* structure of the world involves intersubjectivity, it is generative. The concept of generativity demands that there is a mutual implication of subjectivities that makes it possible for each personal experience to contribute to historical sedimentation, that is handed down to future generations. The process of generativity is infinite and guarantees what Husserl names the “unity of the tradition (Hua/Mat 8,438), as that which keeps communities together; which connects us all to the whole, to which we relate in order to understand ourselves as meaningful beings. Since meaning is socially constituted, it is never absolute, nor final. Over and beyond Heidegger and the finitude of meaning, where that what is meaningful is enclosed in *my* finite life, Husserl’s account of the intersubjective constitution of meaning means that the process is infinite, despite the finitude of my own existence. With the introduction of generativity, the field of meaning is expanded. Before discussing the implications for intersubjective systems theory of the temporal broadening of the field of meaning, let us take a closer look at the way in which unconsciousness is treated by Stolorow and Atwood.

### 1.4 The unconscious as dynamic and situational

Unsurprisingly, Stolorow and Atwood identify a fundamental problem with the Freudian metapsychological determination of unconsciousness, where consciousness would be but the effect of unconsciousness. In contrast to the Freudian unconscious, intersubjective systems theory emphasises *affectivity* as co-constituted within relational dynamics, which amounts to stating that an emotional experience cannot be separated from the intersubjective situation in which it is felt. A good example is the emotional development of a child. Once a child’s emotional experiences are rejected, or mirroring fails, the child must repress the aspects of her affective experience that are unbearable to her caregiver (Stolorow, 2011, 29). Within this context, repression is a “negative organising principle,” which, unlike Freud’s view of repression, is embedded in an intersubjective context, and determines which aspects of a person’s emotional world can be made manifest. Those aspects that cannot be made manifest, remain unconscious, not because they have actively been repressed, but because they could never become articulated “in the absence of a validating intersubjective context” (Stolorow, 2011, 29).

Locating affectivity at the centre of an intersubjectively constituted subjectivity, instead of drives, implies, claim Atwood and Stolorow, a “radical contextualization of virtually all aspects of human psychological life” (Stolorow and Atwood, 2019, 59). Thus: the limits between what can and what cannot be consciously experienced changes within different emotional, and thus relational, contexts. In in other words: intersubjectivity informs the boundaries of what is conscious and unconscious. The changing nature of limits means that Stolorow and Atwood call for a replacement of the Freudian economic tripartition of the Id, Ego, and Superego with a “conception of psychological structures as consisting in the invariant principles, meanings or schemas through which emotional experience comes to be organized according to characteristic schemes and patterns” (Stolorow and Atwood, 2014, 102). These organising principles are produced by the repeated interactions within the developmental systems, and are considered the “building blocks of personality development.” Stolorow and Atwood note that they are unconscious, which, for them, does not mean that there is repressed content in the psyche, but that the organising principles are pre-reflective” (Stolorow and Atwood, 2014, 102).

From the above, it hopefully becomes clear that Stolorow and Atwood thus assign quite an ambiguous role to the Freudian unconscious. They seem, on the one hand, to attempt to save its significance by assuming that feelings can remain unconscious in the sense that they cannot be manifested in an intersubjective context of validation. In other places, they actively call for its replacement: “In place of the Freudian unconscious […], we envision a multiple contextualized experimental world, an organized totality of lived experience, *more or less* conscious” (my Italics) Stolorow (2001).

If this is what is required for intersubjective relations to take precedence over inner mental states, one is left to wonder whether intersubjective systems theory still belongs to the domain of psychoanalysis as the “science of unconscious processes” (Freud, 1961, 70). This question is not merely important for historical or semantic reasons: intersubjective systems theory must be committed to a domain of unknowability, since it is only possible to become aware of something, or for new meaning constellations to be established (in a clinical setting for example), if this meaning can arise out of *somewhere*: a place of hiddenness. Or, in other words: if there were only consciousness, and only meaning, there is no possibility for new meanings to emerge, because meaning cannot be constituted out of a vacuum.

Let us first take a closer look at Freud’s account of the unconscious and clarify what problems Stolorow and Atwood identify in Freud. Here, one should already note that the unconscious is thought in different ways by Freud throughout his life: from a depository of unconscious thoughts to an instance for the active processing of such thoughts. Within the context of Stolorow’s and Atwood’s systems theory, what matters most is that Freud differentiates between the unconscious in a descriptive sense, as that psychical process of which we know nothing at all (Freud, 1990, 69); and the unconscious as “preconscious”: as that which is on the way to becoming conscious experience (again). About the first kind, Freud writes the following:

The unconscious is the true psychical reality; in its innermost nature, it is as much unknown to us as the reality of the external world, and it is as incompletely presented by the data of consciousness as is the external world by the communications of our sense organs (Freud, 1900, 213).

From a phenomenological perspective, Husserl’s critique of Freud mirrors his critique of Kant in the sense that the articulation of an unconscious that is impenetrable by consciousness, amounts to operating from within the natural attitude and is no different from the metaphysical assumption of a thing in itself. Stolorow’s and Atwoods’ comments regarding the Freudian schema echoes Husserl’s critique on Kant: they write that Freud’s repression barrier is conceived in terms of a “fixed intrapsychic structure within an isolated Cartesian container” (Stolorow and Atwood, 2019, 61) and thus reject Freud’s rigid structural separation between on the one hand a representing consciousness, and on the other hand an inaccessible unconscious.

The question then becomes: how is it possible to conceive of an unconscious that moves beyond a naturalistic and causal conception of psychic life, where consciousness would be nothing but a product of unconsciousness? Or, in other words, how to conceive of the unconscious not as a thing in itself but in terms of a dynamic relation, and so that it can play a role in the emergence of meaning? This distinctly phenomenological question necessarily has to deal with the paradoxical situation in which something unconscious can somehow appear in consciousness, without consciousness usurping the foreign element to the presence of a representational consciousness. The first elements of an answer to this question are offered by Husserl, as I seek to make clear below.

Husserlian phenomenological questioning concerns the conditions of possibility of appearance. By elucidating the tacit structures of transcendental constitution, phenomenology, as the term already indicates, points to the metaphor of light: to manifestation, consciousness, clarity, seeing. In the thought of Husserl, any phenomenon is related to an act of consciousness. Yet, when we consider Freud’s unconscious as “preconscious,” we can conceive of it in terms of a problematization of transcendental consciousness in the sense that there is no direct experience of the unconscious, since it is precisely on the way in and out of consciousness as a “point of zero affection,” where consciousness remains passive. It reveals that the acts of transcendental consciousness are based on a domain of indeterminate pre-givenness, which Husserl also describes as a “halo” of non-actual mental processes (Husserl, 1989, 72) and are labelled “confused”:

Every spontaneous act, after being performed, necessarily passes over into a confused state; the spontaneity, or if you will, the activity, to speak of it more properly, passes into a passivity” (Husserl, 1989, 13–14).

Here, two points need to be made with regard to our question: the first is that the halo reveals that consciousness does not only exist in actuality (Lanfredini, 2018, 2); the second is that inactuality is a *modification* of actuality and subordinate to it (Lanfredini, 2018, 2). These elements are reflected in the core of phenomenological questioning: the problem of temporality. Husserl’s treatment of time-consciousness already reveals a certain opacity with regard to a subject’s experience of the passing of time: protention and retention are understood as elements of non-presence, the fading away of this that cannot be remembered or anticipated in terms of a representation. As such, Husserl has already opened the domain of unknowability, hiddenness, lack of the presence of consciousness to itself.^1^ As Lanfredini skilfully clarifies, the dimension that cannot be captured by consciousness, is of affection, which includes “sensibility, what imposes itself, the pre-given, the driven in the sphere of passivity. What is specific therein is motivated in the obscure background” (Husserl, 1989, 234; Lanfredini, 2019, 3). This is where Husserl locates the Freudian unconscious. Not however, as a thing in itself, but still as a thing *of* consciousness, belonging to it, since it can be retrieved by it. Because what is non-present can only be called as such in relation to the present, awareness, consciousness, and can always be recalled, activated and awakened again by consciousness. Or, in other words: non-presence remains subordinated to the privileged position of a consciousness that remains fundamentally present to itself.

A phenomenological treatment of the unconscious thus shows that the Freudian “pre-conscious” defines the place and moment of transcendental determination. Within this context, affectivity and its temporal dimension operates as a limit (there are others too: like death, other people, sleep) case of Husserlian thought in the sense that within conscious experience, there is already reference to something that is not fully given, but a modification of experience, and thus operating at the borders of sense-constitution. In this way, Husserlian phenomenology as a science of consciousness, is also a science of its limits, that are relevant from a transcendental perspective.

Furthermore, phenomenology, as the science concerned with appearance, necessarily deals with hiddenness, although not a hiddenness beyond the limits of what can possibly be experienced. There is no originary or transcendent hiddenness to be found in Husserl’s thought since it is always co-given in experience.^2^ From a phenomenological perspective, what is hidden is situational, and that which allows phenomena to appear in the way they do. Or, in other words: what remains hidden is the dynamic element of the relational essence of any experience. An account of the unconscious as hiddenness that defines transcendental determination, as offered by Husserl, is precisely what Stolorow and Atwood refer to when they describe the unconscious as situational, dynamic and (intersubjective-)context-dependent.

## 2 Discussion

In this paper, I have sought to show that intersubjective systems theory’s transcendental poverty is partly due to its reliance on Heidegger’s Existential Analytic. I have attempted to demonstrate that Husserl offers intersubjective systems theory a phenomenological approach to the intersubjective constitution of meaning. Yet, the need of a philosophically sound phenomenological framework for intersubjective system theory is not only relevant from a theoretical perspective. I think that Husserlian transcendental phenomenology introduces a specific temporal experience that has two fundamental implications for clinical practice, which are mainly outline the scope of this paper, but which I discuss briefly below.

The first point concerns the introduction of a temporality of infinity from a transcendental perspective. As discussed, intersubjective constitution opens the infinity of time. Changing the temporal paradigm of meaning opens up a wide range of transformations of key terms that are in use to describe the clinical encounter. At present, in the literature, this encounter is often described according to themes like empathy (Ratcliffe), solidarity, kinship-in-finitude (Stolorow and Atwood). All these follow from a Heideggerian discourse, and thereby depend on a fundamentally finite temporality, that reveals meaning fundamentally in isolation of others.

The introduction of infinite time to the domain of what is meaningful, opens time, beyond my finite existence, to a future that does not have to be *my* future to nevertheless be meaningful for me during my life. The significance of such a future has been addressed by many theorists working in the phenomenological tradition, in themes like survival and promise (Derrida) and fecundity, pardon and forgiveness (Levinas). I think the departure of a fatalist, isolationist discourse in search for the promise of new meaning opens the clinical dialogue in the sense that, fundamentally, my sense of self is already invested in those who are not me, those who came before me and those who will come after me. Is not that the fundamental import of intersubjectivity?

Secondly, the clarification of the place and role of the unconscious from a phenomenological perspective presents the unconscious as a dynamic and situational limit. It shows, fundamentally, that what I, as a subject, can be conscious of, changes in the sense that my conscious gaze moves. As noted, such an account of consciousness is fundamentally different from Freud’s view of consciousness as an effect of unconsciousness.

Here, it must be noted that Husserl’s thought remains limited in the sense that, in its preoccupation with a phenomenology of consciousness and thus of presence and the possibility of retrieving it, unconscious remains object of consciousness. There are others who, at least in part through their discourse with Husserl, think through the implications of the limitations of consciousness: for example, Merleau-Ponty, who, in describing the relation between the visible and the invisible, does not (like Husserl would) perceive of the invisible as that which is outside the domain of what is visible, but rather as that which fundamentally belongs to what is visible: “pure transcendence without an ontic mask” (Merleau-Ponty, 1968, 229).

However, Husserl’s account of the unconscious lays the actual groundwork for a proper phenomenology of the unconscious in the sense that his phenomenological account of consciousness allows a “way back”: I can turn my gaze to what remained not conscious at first, and can integrate it in my conscious experience. Such a return is a requirement for any kind of therapeutic relation to be possible at all. Here, healing, conceived as going the way back, and committing to the work of memory, finds its original possibility. As Ricoeur (1965) describes, memory does not mean to grasp something out of a place where past experiences are stored, but memory is a *work* of the past: an active and living force. As such, memory is not only limited to what has been, but also regards the present and the future. Going back is a rejection of fatalism: it can change the weight of the past by finding a new relation to it, in the present, and for the future.

## Author contributions

RU: Writing – original draft.

## Footnotes

^1^This is a question addressed by Bernet (2002): his insightful Freudian reading of Husserl’s *Phenomenology of Inner Time Consciousness* offers a phenomenological grounding of the Freudian unconscious that offers a possibility of a conscious and symbolic representation of the drives, while also criticizing the metaphysical elements of the Freudian unconscious.

^2^As the most classical example of Husserl’s approach to hiddenness can serve his remark in *Ideas 1* that “a physical thing can be given only “one-sidedly” (*Ideas*, 1989, 94).
